# Revealing the coexistence of differentiation and communication in an endemic hare, *Lepus yarkandensi*s (Mammalia, Leporidae) using specific-length amplified fragment sequencing

**DOI:** 10.1186/s12983-021-00432-x

**Published:** 2021-09-26

**Authors:** Buweihailiqiemu Ababaikeri, Yucong Zhang, Huiying Dai, Wenjuan Shan

**Affiliations:** 1 Xinjiang Key Laboratory of Biological Resources and Genetic Engineering, College of Life Science and Technology, Xinjiang University, Urumqi, 830046 China; 2 College of Xinjiang Uyghur Medicine, Hoten, 848000 Xinjiang China

**Keywords:** Yarkand hare, Specific-length amplified fragment sequencing (SLAF-seq), Genetic differentiation, Genetic diversity, Gene flow, Adaptation, Tarim Basin

## Abstract

**Background:**

The Yarkand hare (*Lepus yarkandensis* Günther, 1875) is endemic to oasis and desert areas around the Tarim Basin in the Xinjiang Uyghur Autonomous Region of northwest China; however, genome-wide information for this species remains limited. Moreover, the genetic variation, genetic structure, and phylogenetic relationships of Yarkand hare from the plateau mountain regions have not been reported. Thus, we used specific-length amplified fragment sequencing (SLAF-seq) technology to evaluate the genetic diversity of 76 Yarkand hares from seven geographic populations in the northern and southwestern parts of the Tarim Basin to investigate single-nucleotide polymorphism (SNP) marker-based population differentiation and evolutionary processes. Selective sweep analysis was conducted to identify genetic differences between populations.

**Results:**

Using SLAF-seq, a total of 1,835,504 SNPs were initially obtained, of which 308,942 high-confidence SNPs were selected for further analysis. Yarkand hares exhibited a relatively high degree of genetic diversity at the SNP level. Based on pairwise *F*_ST_ estimates, the north and southwest groups showed a moderate level of genetic differentiation. Phylogenetic tree and population structure analyses demonstrated evident systematic phylogeographical structure patterns consistent with the geographical distribution of the hares. Hierarchical analysis of molecular variation further indicated that genetic variation was mainly observed within populations. Low to moderate genetic differentiation also occurred among populations despite a common genomic background, likely due to geographical barriers, genetic drift, and differential selection pressure of distinct environments. Nevertheless, the observed lineage-mixing pattern, as indicated by the evolutionary tree, principal component analysis, population structure, and TreeMix analyses, suggests a certain degree of gene flow between the north and southwest groups. This may be related to the migration of hares to high-altitude water sources southwest of the basin during glacial climatic oscillations, as well as river re-diffusion and oasis restoration in the basin following the glacial period. We also identified candidate genes, and their associated gene ontology terms and pathways, related to the adaptation of Yarkand hares to different environmental habitats.

**Conclusions:**

The identified genome-wide SNPs, genetic diversity, and population structure of Yarkand hares expand our understanding of the genetic background of this endemic species and provide valuable insights into its environmental adaptation, allowing for further exploration of the underlying mechanisms.

**Supplementary Information:**

The online version contains supplementary material available at 10.1186/s12983-021-00432-x.

## Background

Identifying the levels of genetic variation within and between species or populations is an essential step in studying the influences of mutation, natural selection, and genetic drift [1]. Toward this end, it is often beneficial to understand genetic variation using population differentiation statistics such as the pairwise genetic differentiation estimate (*F*_ST_) [2]. Population differentiation is a significant step toward speciation [3], potentially leading to the formation of new species or subspecies. The extent of genetic differentiation is shaped by various correlated and interacting factors, including population and migration sizes, breeding and mating systems, dispersal barriers, gene flow, social behaviors, reproductive strategies, and ecological selection structures [3]; among these factors, gene flow is the most important determining factor for genetic structure and differentiation in wild populations [4]. Furthermore, environmental factors may influence the colonization process, potentially affecting gene flow. Disruptions in dispersal processes, such as physical obstacles to migration, exchange of individuals among wildlife populations, and increased inbreeding within spatially isolated populations can reduce gene flow, leading to genetic differentiation [5, 6]. To date, research investigating the factors influencing genetic differentiation and gene flow within a species has mainly focused on geographical or geological factors—such as the impact of Quaternary glacial fluctuations [7–9] and habitat fragmentation [10, 11]—combined with anthropogenic activities, resulting in physical barriers that cause discontinuities in the distribution of a species [12].

The Yarkand hare species *Lepus yarkandensis* Günther, 1875 is distributed across marginal oases along the edges of rivers in the Tarim Basin, southern Xinjiang Uygur Autonomous Region (XUAR), northwest China [13]. The Yarkand hare relies on vegetation near streams that flow down from the melting water of surrounding snowy mountains. Its habitat includes poplar forests and brushwood along the river margins, and its distribution is restricted to riverine patches and scattered oases at altitudes between 900 and 1200 m; these oases are physically isolated by the Taklamakan Desert [13, 14]. Kumar et al. [8] suggested that mountain habitats may also be suitable for Yarkand hare in the face of ongoing climate-induced range expansion. Indeed, our field investigations showed that the Yarkand hare is distributed in the mountain areas of Tashkurgan, Aketu, and Wuqia in the Pamir Plateau southwest of the Tarim Basin. The Yarkand hare shows strong adaptability to the extreme aridity, intense solar radiation, and intense heat of the Tarim Basin [15], which underwent desertification 5.3 million years ago (Mya) [16]. Over the past decade, wild populations of this species have drastically declined due to habitat fragmentation and deterioration of their distribution area resulting from aggravated human activities, including local economic development, oil exploitation, and illegal hunting. Consequently, the Yarkand hare is listed as a “vulnerable species” on the China Species Red List [17], and is now listed as “near threatened” by the International Union for Conservation of Nature [18].

Resolving the phylogenetic relationships between species and different populations within a species is a very important task in evolutionary biology and conservation genetics [6]. Previous studies exploring the genetic variation and phylogenetic relationships of Yarkand hare populations have focused on mitochondrial DNA (mtDNA) genes [8, 15, 19–21], the male-specific Y-chromosomal sex-determining region (*SRY*) gene [21], and two nuclear DNA (nDNA) markers, namely, the mechano-growth factor (*MGF*) and spectrin beta non-erythrocytic 1 (*SPTBN1*) genes [8]. Phylogenetic analysis of mtDNA sequences showed significant genetic differentiation among most Yarkand hare populations, highlighting low migration levels among populations inhabiting oases isolated by the Taklamakan Desert. This barrier proved to be effective against gene flow, suggesting the importance of habitat aridification, oasis development, and river runoff in the differentiation and evolutionary history of Yarkand hare populations [19, 20]. However, these studies were limited by only analyzing mtDNA and nDNA fragment markers, and failed to include populations living in plateau mountain regions.

To the best of our knowledge, a systematic genome-wide investigation of Yarkand hare genetic diversity, population structure, and phylogenetic relationships has not yet been conducted. Next-generation sequencing technology enables the identification of a large number of markers, including single-nucleotide polymorphisms (SNPs), across the genome in a cost-effective and highly reproducible manner. Given its high success rates, specificity, stability, low cost, and labeling efficiency, specific locus amplified fragment sequencing (SLAF-seq) can be directly used for chromosome-specific molecular marker development without the need to sequence the entire genome of a species. Indeed, SLAF-seq has been successfully used for gene identification [22] as well as in analyses of the genetic diversity and phylogenomics of several species [23–25]. Genomic data analysis provides detailed information on a population’s genetic variations, historical dynamics, and adaptive characteristics, which can expand knowledge of genomes for non-model species, enabling comprehensive evaluation of evolutionary patterns and signatures that may benefit conservation efforts.

Species with a high level of population differentiation and a limited distribution range among populations may have reduced ability to cope with adverse environmental conditions [26, 27]. If a local population disappears or decreases, a large proportion of the total genetic variation may be lost [28]. These populations may then become more vulnerable to random genetic drift, which may contribute to population differentiation by randomly fixing alleles. Moreover, geographic isolation coupled with characteristics of a small population size and local adaptation leads to reduced genetic variation due to a decrease in gene flow [28]. Therefore, the extant populations of a species result from an often complex demographic history involving population splits, gene flow, and population size changes. Accurate data on the geographic boundaries of isolated populations, and the degree of genetic differentiation and individual exchange among these populations are thus vital for accurate species risk assessments and effective conservation planning [30]. Moreover, the detection of genomic differences can shed light on the genetic basis of adaptation to diverse environments and provide insights into functionally important genetic variants [31].

Therefore, to establish effective protection measures and sustainable management of Yarkand hare genetic resources in Xinjiang, China, we used the SLAF-seq approach to identify genome-wide SNP markers in Yarkand hare populations for the first time. Based on these data, we investigated the genetic diversity and differentiation, migration events, and evolutionary process of diversification of these populations. We also sought to identify possible genomic signatures of adaptation to various environmental conditions found across the range of this species by sampling individual hares from the northern and southwestern regions of Tarim Basin. Our specific research questions were as follows: (i) what is the genetic variation, differentiation, and phylogenetic relationship of Yarkand hare populations at the genome-wide level? (ii) What is the historical pattern of divergence and gene flow between populations? (iii) Are there genomic differences that may be related to environmental stress or their adaptation? These findings can help to provide a comprehensive view of the genetic structure and relationships among Yarkand hare populations and shed light on genomic regions that harbor genes related to adaptive traits in this species.

## Materials and methods

### Sampling and DNA extraction

**Fig. 1:**
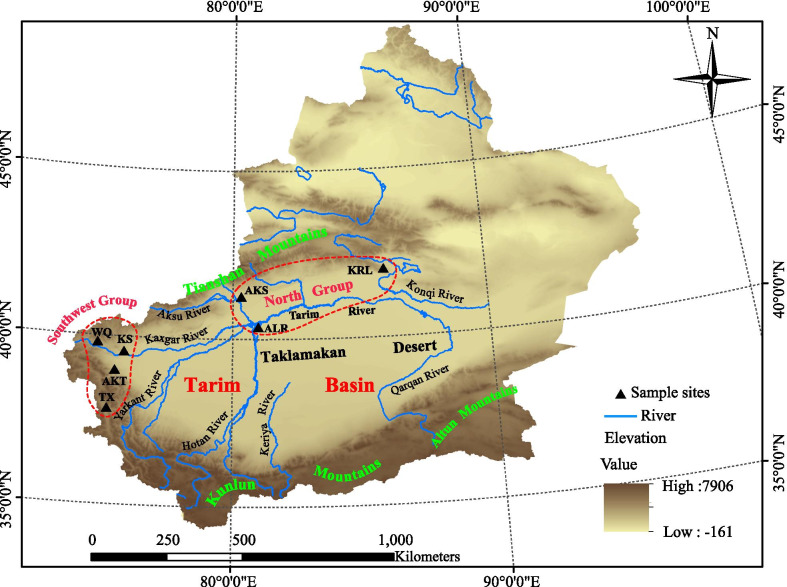
Approximate sampling sites of Yarkand hare populations in Xinjiang, China

Muscle or skin tissue samples were collected from a total of 76 Yarkand hares (*L. yarkandensis*) from seven geographic populations around the Tarim Basin from 2008 to 2018; 20 samples were obtained from Korla (KRL), 10 from Akesu (AKS), 5 from Alar (ALR), 12 from Tashkurgan (TX), 16 from Aketu (AKT), 10 from Kashgar (KS), and 3 from Wuqia (WQ) in XUAR, northwestern China. Some samples were obtained from roadkill or hares that died of natural causes, whereas others were obtained from specimens that had been confiscated from illegal poachers (provided by local forestry bureaus). The geographical details of the sampled populations are shown in Fig. 1. For ease of analysis, we divided these populations into two groups (north and southwest) based on their geographical location in the Tarim Basin. The north group included AKS, ALR, and KRL; these hare populations reside in the middle and lower reaches of rivers, where the climate is relatively hot and arid with an elevation not exceeding 1500 m. The southwest group included the TX, AKT, KS, and WQ populations that live in an environment characterized by drought in the mountain areas of the Pamir Plateau along the upper reaches of Tarim River with an elevation higher than 1500 m, even reaching up to 3000 m in some areas, including TX.

Muscle samples were preserved in sterile tubes with anhydrous alcohol at –80 °C until total genomic DNA extraction using the standard phenol–chloroform extraction protocol [32]. Genomic DNA integrity was determined using 1.0% agarose gel electrophoresis with a lambda DNA standard, and the DNA purity and concentration were assessed using the Nanodrop ND-1000 spectral photometer (NanoDrop, Wilmington, DE, USA). A minimum of 30 ng/μL of DNA was used for sequencing.

### SLAF library preparation, sequencing, and quality control

We used the domestic rabbit (*Oryctolagus cuniculus*) OryCun 2.0 genome [33], downloaded from the National Center for Biotechnology Information (NCBI; ftp://ftp.ncbi.nlm.nih.gov/genomes/all/GCF/000/003/625/GCF_000003625.3_OryCun2.0/) for a pre-experiment in silico simulation of the number of markers produced by multiple endonuclease combinations; the optimal restriction enzyme combination was predicted to be RsaI-EcoRV-HF®. Based on the results of the pilot experiment, we constructed the SLAF library as per the methods reported by Sun et al. [34] and Zhang et al. [35], with slight modifications; DNA fragments 314–344 base pairs (bp) in size were selected as SLAFs and used for paired-end sequencing on an Illumina HiSeq 2500 system (Illumina, Inc., San Diego, CA, USA) at Beijing Biomarker Technologies Corporation (Beijing, China). Enzyme digestion efficiency, fragment insertion distribution, and alignment efficiency of the positive control (*Oryza sativa* ssp. *japonica*, http://rapdb.dna.affrc.go.jp/) were calculated using SOAP v2 software [36] to evaluate the reliability of the enzyme digestion experiment and the accuracy of library construction.

We conducted real-time monitoring for each cycle during sequencing to control the quality of the sequencing data. We also calculated the ratio of high-quality reads according to raw read quality scores greater than Q30 (i.e., a quality score of 30, indicating that the probability of error is 0.1%, and thus providing 99.9% confidence) and the guanine-cytosine (GC) content as the two key quality indicators. Burrows-Wheeler Aligner (BWA) v0.7.5a-r405 [37] was used to map all sample reads onto the OryCun 2.0 genome sequence.

### Developing SLAF tags and SNP markers

SLAF tags were mined according to the fragment size defined by the enzyme digestion scheme; SAMtools v0.1.18 [38] and GATK v3.3.2 [39] were used for SNP calling following evaluation of the sequencing depth and SLAF tag distributions on chromosomes. A locus was defined as a reliable SNP if it was simultaneously called by both packages. Filtering of high-quality SNPs was conducted according to the criteria of site information integrity (INT) ≥ 0.5 and minor allele frequency (MAF) ≥ 0.05 using Plink v1.07 software [40]. Finally, the selected high-quality SNPs were used for further analysis.

### Genetic diversity analysis

Summary statistics of genetic diversity, including nucleotide diversity (*π*), observed heterozygosity (*H*_*o*_), expected heterozygosity (*H*_*e*_), and the polymorphism information content (*PIC*), were calculated using Arlequin ver3.5 [41] and Power-Marker v3.25 [42].

### Phylogenetics, population structure, and principal component analysis

Phylogenetic analyses were conducted using Bayesian inference (BI) and maximum-likelihood (ML) trees of an individual SNP matrix to clarify the phylogenetic relationships among geographic Yarkand hare populations from a genome-wide perspective; the rabbit (*O. cuniculus*) genome was used as the outgroup. BI phylogenetic analysis was performed using Exabayes ver1.4.1 [43]; 5 million generations were used for Bayesian Markov chain Monte Carlo (MCMC) iterations, with sampling every 500 generations. We performed ML analysis using IQ-TREE v1.6.1 [44], with 100 bootstraps to test the confidence of the branches. ModelFinder [45] was used to determine the best-fit base pair substitution model according to the Bayesian information criterion (BIC); the general time-reversible (GTR) model was selected as the optimal model for our datasets. The final BI tree was visualized and edited in FigTree ver1.4.2 (http://tree.bio.ed.ac.uk/software/figtree).

We estimated the pairwise population genetic fixation index *F*_ST_ and performed hierarchical analysis of molecular variation (AMOVA) using Arlequin ver3.5; the significance of variance components was determined via 10,000 random permutations. Principal component analysis (PCA) of all samples was conducted using EIGENSOFT ver7.2.1 [46], with the first three eigenvectors of total variation visualized. The population structure among the studied Yarkand hare populations was inferred using ADMIXTURE ver1.22 [47] with a Bayesian clustering method. Based on the same set of SNPs, the number of subgroups (*K*) predicted from 1 to 10 and the number of ancestors was determined according to the position of the minimum value with an error rate obtained from cross-validation (CV) [48].

### Divergence time estimation and gene flow analysis

A relaxed molecular clock model based on the ML method was used to estimate the divergence time using the MCMC Tree program in PAML ver4 [49]. Two points were calibrated to build the time tree, (1) the split of the genus *Lepus* occurring approximately 10.76 Mya (± 0.86 Mya), and (2) the divergence time of Yarkand hare estimated at approximately 0.64 Mya (± 0.26 Mya), as established by previous work [50] that used time constraints from data published by Matthee et al. [51] and numerous fossil records were used to calibrate the evolutionary time of the genus *Lepus* and divergence of the Yarkand hare. The MCMC analysis was performed for a total of 1 × 10^6^ generations with sampling every 50 generations; the first 5% of the trees were discarded as burn-ins. To estimate historical gene flow between populations, TreeMix ver1.12 [52] was used to deduce the pattern of population differentiation and mixing based on genome-wide allele frequencies in the branch graph, which best describes the relationship between populations and gene flow.

### Selection sweep, gene annotation, and functional analysis

We calculated the locus-specific divergence in allele frequencies between Yarkand hare populations and their different environment counterparts sampled in this study, based on the unbiased estimates of pairwise *F*_ST_ using VCFtools [53]. To detect genomic regions related to the selection of hare populations in different environments, we calculated all possible pairwise *θ*π values, which were log_2_-transformed (π_control group_/π_different environment group_). Both *F*_ST_ and *θ*π values were quantified using a non-overlapping 10-kb sliding-window approach. Genes overlapping either partially or entirely within the 95% threshold of the empirical distribution of the raw *F*_ST_- values and log_2_ (*θ*π ratio) within regions were considered putative selection genes. Functional annotation for each candidate gene in these target regions was identified by comparing the gene set against the Kyoto Encyclopedia of Genes and Genomes (KEGG) database using the NCBI BLASTp tool [54]. Gene Ontology (GO) terms were obtained using Blast2GO [55] with default parameters. Metabolic pathways involving these genes were identified using the KEGG Automatic Annotation Server (KAAS) [56] with the bi-directional best-hit method. GO term and KEGG pathway enrichment analyses were then performed using the ClusterProfiler R package [57]. We applied a raw *p*-value cut-off of < 0.05 to identify significantly enriched GO terms and KEGG pathways.

## Results

### High-throughput SLAF sequencing

DNA sequences of 314‒344 bp were selected as SLAFs for the Yarkand hare, and approximately 215, 273 SLAF tags were projected to be produced. Sequencing results for the positive control indicated that enzyme digestion efficiency, comparisons, and fragment selection evaluations were normal and reliable.

High-throughput sequencing of the SLAF library yielded 373.19 Mb raw reads, with an average of 4,910,380 reads per individual. After strict filtration, 372.14 Mb of high-quality clean data, with an average of 4,896,576 reads per individual, were obtained (Additional file 1: Table S1). Furthermore, the average Q20 and Q30 was 98.12% and 95.43%, respectively (Additional file 1: Table S1), indicating the reliability of the tested sequence results. The average mapping rate of our samples to the reference genome (OryCun 2.0) was 95.16% and the average GC content was 41.29% (Additional file 1: Table S1).

### Development of SLAF tags and SNP marker selection

A total of 3,527,350 SLAF tags were generated from the 76 specimens, with an average sequencing depth of 13.95 × (Additional file 1: Table S1), which were well distributed across all chromosomes (Additional file 2: Fig. S1). A total of 1,835,504 SNPs were identified across all samples following alignment to the reference genome, and the SNP integrity ranged from 31.38% to 47.38%, with an average of 39.84% (Additional file 1: Table S1). To reduce the sequencing errors, baseline differences were removed and accuracy was assessed, resulting in 308,942 highly consistent and confident SNP markers (MAF ≥ 0.05 and INT ≥ 0.5) that were selected for further analysis.

### Genetic diversity and differentiation

Nucleotide diversity (*π*) ranged from 0.0524 (KRL population) to 0.0845 (TX population) across the seven geographic populations of Yarkand hare, with an average of 0.0655 per population (Table 1). The average *H*_*e*_, *H*_*o*_, and *PIC* values of all populations were 0.3130, 0.2582, and 0.2543, respectively, with the highest and lowest values observed in the WQ and KRL populations, respectively (Table 1). In total, excluding the AKT population, the genetic diversity indices of the southwest group were higher than those of the north group (Table 1).

**Table 1:** Summary statistics of the genetic diversity of Yarkand hares analyzed in this study

Group	Population (abbreviation)	Number of samples (N)	Nucleotide diversity (*π*)	Expected heterozygosity (*He*)	Observed heterozygosity (*Ho*)	Polymorphism information content (*PIC*)
North group	Akesu (AKS)	10	0.0551	0.3060	0.2380	0.2493
	Alar (ALR)	5	0.0592	0.3376	0.3143	0.2733
	Korla (KRL)	20	0.0524	0.2659	0.2030	0.2186
Southwest group	Taxkorgan (TX)	12	0.0845	0.3030	0.2451	0.2464
	Aketu (AKT)	16	0.0543	0.2744	0.2207	0.2266
	Kashgar (KS)	10	0.0835	0.3194	0.2294	0.2595
	Wuqia (WQ)	3	0.0697	0.3846	0.3566	0.3065
Mean		10.86	0.0655	0.3130	0.2582	0.2543

Samples were divided into two groups based on geographic location on the Tarim Basin

Estimated *F*_ST_ values (Table 2) among all pairs of populations were generally low to moderate, ranging from 0.0161 to 0.1297, indicating the presence of genetic structuring among these Yarkand hare populations. Moderate differentiation was noted between the southwest KS and WQ populations and all north populations, whereas only minimal genetic differentiation was found between the southwest AKT and TX populations and all north populations. Notably, the differentiation degree (*F*_ST_ = 0.0689–0.1297) between TX and the other southwest populations (KS, WQ, and AKT) was higher than that between TX and the north group populations (*F*_ST_ = 0.0472–0.0633) (Table 2), even though the TX population is geographically located in the southwest region of the Tarim Basin.

**Table 2:** Pairwise *F*_ST_ values among different geographic populations of Yarkand hares

Population	AKS	ALR	KRL	TX	AKT	KS	WQ
AKS							
ALR	0.0501						
KRL	0.0161	0.0392					
TX	0.0570	0.0633	0.0472				
AKT	0.0382	0.0520	0.0283	0.0689			
KS	0.1029	0.1052	0.0918	0.1297	0.0448		
WQ	0.0932	0.1027	0.0832	0.1223	0.0470	0.0608	

Genetic differences among the seven geographical populations were further examined using AMOVA, suggesting that the genetic differences predominantly originated from within-population differences (90.66%, *p* < 0.01); only 9.34% of the variability was partitioned among populations (*p* < 0.01) (Table 3). When pooling individuals into two to three groups based on their geographic distribution in the Tarim Basin (according to the *F*_ST_ results, the southwest TX population was included in the north group or separated as its own group), the genetic variation within populations was much higher than that among groups or populations.

**Table 3:** AMOVA among the Yarkand hare populations

Groups	Differentiation coefficient			Percentage of variation (%)		
	*F*_CT_	*F*_SC_	*F*_ST_	Between groups	Between populations	Within population
[AKS; ALR; KRL; TX; AKT; KS; WQ]	–	–	0.093**	–	9.34**	90.66
[AKS; ALR; KRL; TX] [AKT; KS; WQ]	0.064**	0.058**	0.118**	6.39**	5.41**	88.20**
[AKS; ALR; KRL] [TX] [AKT; KS; WQ]	0.070*	0.048**	0.115**	6.98 *	4.49**	88.54**

*F*_CT_ represents genetic differences among groups; *F*_SC_ represents genetic differences among populations within groups; *F*_ST_ represents genetic differences among populations; * 0.010 < *p* < 0.050, ** 0.010 < *p* < 0.001

### Phylogenetic analysis and population genetic structure

**Fig. 2:**
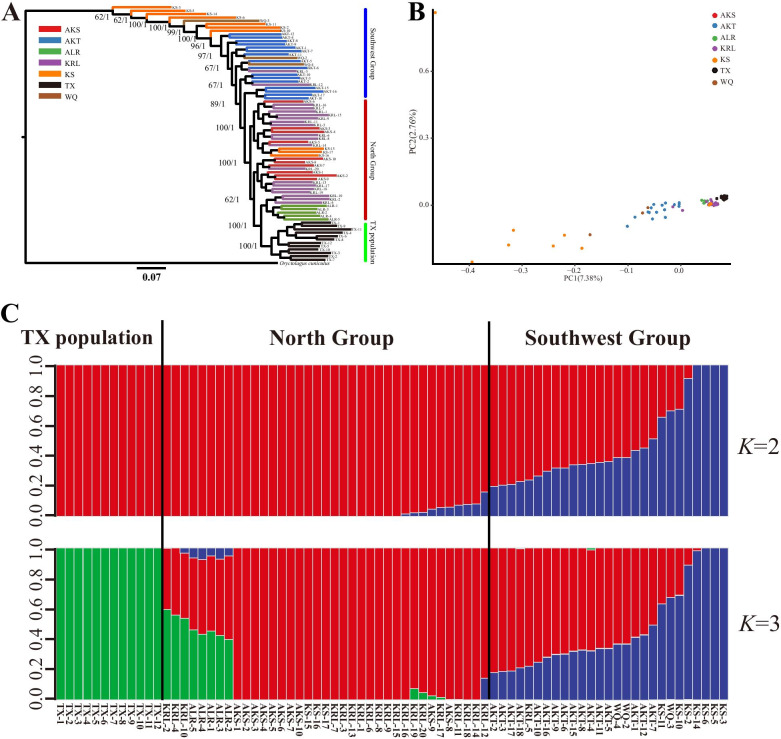
Phylogenetic analyses and population structure of Yarkand hare based on high-quality SNP datasets. **a** Phylogenetic tree with *Oryctolagus cuniculus* as the outgroup. **b** Scatterplot of the principal components for the seven geographic populations. **c** Genetic structure of 76 Yarkand hares. The number of ancestral populations (*K* = 2–3) is indicated

As the topological structure of the BI and ML evolutionary trees was consistent (Additional file 3: Fig. S2), we combined the trees. The Yarkand hares analyzed in this study were divided into two main clusters with high confidence (Fig. 2a). The first branch was predominantly located at the root of the tree, which comprised individuals from the southwest group (WQ, AKT, and KS populations) and two individuals from the KRL population in the north group. The other branch included samples from the north KRL, AKS, and ALR populations; all TX samples; and three individuals from the KS population in the southwest group. Notably, all samples from the TX population in the southwest group clustered with samples from the north group; together, these samples formed the second-largest branch, which included three smaller branches. However, the TX population formed a small branch, fully distinct from the first main cluster comprising the other southwest group samples (Fig. 2a).

Genetic differentiation among the populations was also evident in the PCA (Fig. 2b). Population relationships in the ordination space were largely consistent with the geographical distribution of samples, which was in agreement with the phylogenetic tree (Fig. 2a). Specifically, KS samples were scattered on the left of the PCA plot, whereas almost all other samples were relatively concentrated on the right of the plot (Fig. 2b); the TX population clustered near the north group samples to the far right.

Assessment of the population structure using ADMIXTURE indicated two main ancestral subgroups based on the lowest cross-validation errors at a *K* value of 2 (Additional file 4: Fig. S3). Four KS individuals from the southwest group formed an ancestral cluster when *K* = 2. The majority of the north group samples, all TX samples, and three KS individuals formed another ancestral cluster; the remaining samples from both the southwest and north groups showed different degrees of mixed ancestry (Fig. 2c). However, when *K* = 3, the TX population was further separated, showing a distinct ancestry, whereas all ALR samples and one KRL sample from the north group were mixed among three ancestral clusters (Fig. 2c).

### Divergence time estimation and gene exchange analysis

**Fig. 3:**
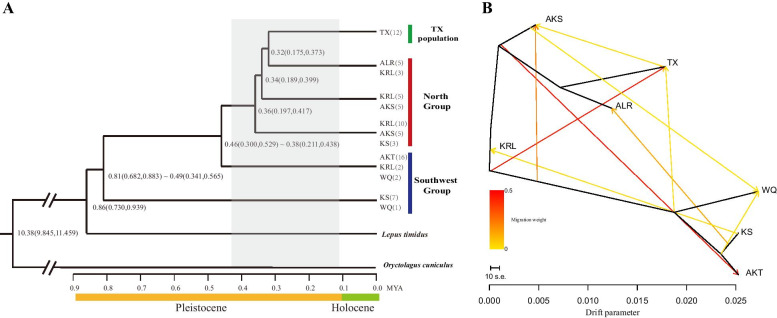
Divergence time and migration events among Yarkand hare populations. **a** Time scale of major divergence in the Yarkand hare populations. The colors of the bars at the branch tips correspond to the group colors in Fig. 2. **b** Inferred Yarkand hare phylogenetic tree with mixture events among populations. Arrows indicate migration events and are colored according to their migration weight. Horizontal branch lengths are proportional to the amount of genetic drift estimated among populations

The MCMC algorithm with a general time-reversible model base pair substitution model was used to construct a Yarkand hare differentiation time-merging tree (Fig. 3a). The Yarkand hare’s most recent common ancestor, *Lepus timidus*, occurred approximately 0.86 Mya [95% highest posterior density (HPD): approximately 0.73–0.94 Mya]. The Yarkand hare population differentiation events predominantly occurred approximately 0.81–0.32 Mya, exhibiting a general south-to-north trend, with the southwest KS population showing the earliest differentiation (0.81 Mya).

We inferred Yarkand hare migration events using TreeMix to further reveal the population structure with ADMIXTURE (Fig. 3b). Consistently, the tree topology indicated that all of the southwest populations, excluding TX, clustered into a single branch, whereas the north group populations and the southwest TX population clustered into a separate branch. A large number of migration events were identified among these Yarkand hare populations. Two migrations were detected with strong migratory signals, including one event that began from the north AKS population to the southwest AKT population (migration weight 0.455) and one that began from an ancestor common to both the southwest and north groups toward the southwest TX population (migration weight 0.450).

### Screening for selective sweeps in the Yarkand hare populations

**Fig. 4:**
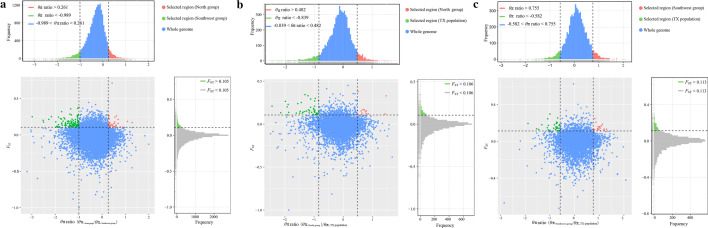
Distribution of *θ*π ratios and *F*_ST_ values. The values were calculated over a 10-kb non-overlapping sliding window. Data points located to the left and right of the left and right vertical dashed lines, respectively (corresponding to the 5% left and right tails of the empirical θπ ratio distribution), and above the horizontal dashed line (corresponding to the 5% right tail of the empirical *F*_ST_ distribution) were identified as selected regions for **a** the southwest group (green points) and north group (red points) populations; **b** the TX population (green points) and north group populations (red points); and **c** the TX population (green points) and southwest group (red points) populations, respectively

Based on the ADMIXTURE and PCA results, as well as the climate and geographical features of the sampling sites, we combined the north group populations (AKS, ALR, and KRL), represented by an arid, hot, and plain environment, into a single gene pool. We combined the southwest group (AKT and WQ), represented by an arid and medium-altitude (elevation between 1500 and 2500 m) environment, into another gene pool. The TX population in the cold, arid, and high-altitude (elevation > 3000 m) environment was considered as a separate group. As the KS population includes samples collected from both plain and medium-altitude (> 1500 m) areas, we excluded this population from this part of the analysis. Selection sweep analyses were performed by calculating the 5% highest *F*_ST_ values and the *θ*π ratio cut-off values between the following group pairs: north group vs. southwest group, north group vs. TX, and southwest group vs. TX, which identified 30, 17, and 15 candidate genes, respectively (Additional file 5: Table S2 and Fig. 4). The functions of these genes—based on their enriched functional GO categories and KEGG pathways—were mainly related to energy metabolism, cell survival and proliferation, water reabsorption, response to stimulus, and oxidative stress (Additional file 6: Table S3, Additional file 7: Table S4, and Additional file 8: Table S5).

## Discussion

In Xinjiang, Yarkand hares inhabit both natural and artificial oases, and are also found along the major river edges in the Tarim Basin encircling the Taklamakan Desert, which is the second largest sand sea on Earth. In this first genome-wide study of the Yarkand hare using SLAF-seq, we found low to moderate, yet significant, genetic differentiation between populations despite relatively long geographic distances. Interestingly, some genetic exchange among populations was also detected in the phylogenetic tree, PCA, and ADMIXTURE analyses. Indeed, the TreeMix analysis estimated a certain degree of gene flow among geographically separated populations. These results indicate that both genetic differentiation and gene exchange co-occur among the populations of this species. In addition, some selection signatures corresponding to genomic regions potentially under selection were identified in association with environmental differences, indicating a certain degree of local adaptation.

### Genetic diversity analyses using Yarkand hare whole-genome SNPs

Genetic diversity in both endangered and common species may play an important role in ecosystem integrity and sustainability, and is also essential for conserving a population’s evolutionary potential to adapt to changing environments [6]. In this study, a set of genome-wide SNP markers was employed for genetic variation analyses. In contrast to previous studies reporting that domestic animals, such as rabbits [58, 59], dogs [60], pigs [61], and horses [62] are less genetically diverse than their wild counterparts, the average genetic diversity indices *π* (0.0655), *PIC* (0.2543), and *H*_*o*_ (0.2582) of the Yarkand hare were slightly lower than those of the domestic rabbit (*O. cuniculus*) [63]. However, this finding is in line with the hypothesis that among mammalian species, rabbits have relatively high levels of nucleotide diversity, which is likely related to their greater long-term effective population size compared with those of other sequenced mammalian species [59, 64]. In addition, the *H*_*e*_ was higher than the *H*_*o*_ in all Yarkand hare populations sampled in this study. A similar heterozygosity pattern was also observed in *Gazella subgutturosa* populations sampled in Xinjiang [65], which may indicate limited genetic exchange among populations possibly caused by natural physical barriers and habitat fragmentation.

The nucleotide diversity of Yarkand hares (*π* = 0.0655) in our study based on whole-genome SNP sequencing was much higher than that previously reported for two mtDNA genes (*π*_*Cytb*_ = 0.008 ± 0.004 and *π*_*D-loop*_ = 0.031 ± 0.015) [15, 20], the male-specific *SRY* gene (*π* = 0.000123) [21], and two nDNA fragment markers (*π*_*MGF*_ = 0.018 and *π*_*SPTBN1*_ = 0.003) [8], confirming that genome-wide SNPs obtained using SLAF-seq can detect abundant variations. Furthermore, the genetic diversity of the southwest group (excluding AKT) was relatively higher than that of the north group, indicating a certain degree of inbreeding in northern populations. One possible explanation for this pattern is that the southwest Tarim Basin—the origin of basin rivers—was a glacial refugium for the Yarkand hare during Quaternary climatic fluctuations [15], providing a suitable environment for maintaining the relatively high genetic diversity of this species. In contrast, rivers in the northern and eastern parts of the basin dried out during the glacial period; thus, flora and fauna depending on oases produced by meltwater either died or retreated to the southwest [15]. Furthermore, as industry and transportation in the southwest Tarim Basin are less developed than those in the north, hare habitats in the southwest are less disturbed by human interference, thereby facilitating frequent gene exchange to ultimately maintain higher genetic variation. In addition, the northern and eastern Tarim Basin ecosystems are more susceptible to habitat loss and degradation than those in the southwest [15]. Habitat degradation caused by fragmentation may have influenced the genetic diversity of the north group populations through reduced gene flow and increased inbreeding. Among them, the KRL population distributed in the northernmost part of the Tarim Basin exhibited the lowest genetic variation level, which may be explained—to some degree—by the geographical isolation and disturbed natural habitats resulting from frequent anthropogenic activities such as increased transportation and expansion of agricultural land.

### Co-occurrence of genetic differentiation and gene flow in the Yarkand hare

Our phylogenetic tree and PCA results showed that when the seven populations were divided into the north (AKS, ALR, and KRL) and southwest (TX, AKT, KS, and WQ) groups or when TX samples from plateau mountain areas were considered as a distinct group (TX population), a clear Yarkand hare phylogeographical distribution pattern was observed. This structure was also supported by the pairwise *F*_ST_ values among populations (Table 2), which indicated moderate genetic differentiation between the southwest KS and WQ populations, and the north group populations, as well as the TX population. Genetic differences among populations in Yarkand hare were further identified via ADMIXTURE with two main distinct lineages (*K* = 2, Fig. 2c) and according to the AMOVA showing significant *p*-values for *F*_ST_ (Table 3). This finding is consistent with that of previous studies showing mitochondrial fragment-based genetic and geographic differentiation between the southwest and northeast Yarkand hare populations [15, 19, 20, 66]. This genetic differentiation pattern among populations also corresponds with evidence regarding morphological differences between hares in the southwestern and northern regions of the Tarim Basin [67]. We speculate that existing geographical barriers physically isolated populations from dispersion and exchange, leading to genetic differentiation. Specifically, the Yarkand hare populations have likely undergone genetic differentiation as a result of irreversible habitat fragmentation [8] and vegetation cover destruction due to anthropogenic actions that have directly changed the course of Tarim River [68] as well as the oasis landscape and vegetative cover over the past 200 years [69]. Regional aridification, shifting sands, and winds have destroyed vast oasis areas in the southern regions of the desert [69, 70], potentially affecting genetic admixture between geographically isolated populations. Reportedly, populations with reduced size in isolated habitats may have differentiated via selection and genetic drift during glacial cycles [71]. Indeed, genetic drift may partly contribute to the differentiation of Yarkand hare [8]. We also found a substantial increase in drift in the other three southwest group populations (i.e., KS, WQ, and AKT) compared with the TX population, as revealed by the TREEMIX results (Fig. 3). Genetic drift may also account for the higher degree of genetic differentiation found between TX and the other southwest populations compared with that found between TX and the north populations. Given that the TX population is geographically located in the southwest of the Tarim Basin, the identified population structure might also reflect the different environmental conditions across the Tarim Basin (i.e., temperature and altitude), which may impose different types of selective pressure on Yarkand hares, as observed for other species such as *Diptychus maculates* [24] and urchins [72] according to genome-wide SNP-based analysis. Future studies should include historical demographic events such as range expansions and population bottlenecks in such analyses, which may shape allele frequency patterns between populations, to explore these hypotheses based on genome-wide SNP markers.

Environmental changes during glacial periods may also merge previously isolated populations. Repeated signatures of migration and mixing are evident in the history of Yarkand hare populations [8, 19]. Despite clear population differentiation and significant pairwise *F*_ST_ values among populations, our phylogenetic analyses also revealed a high degree of lineage admixture (Table 3, Figs. 2 and 3). Population differentiation and mixing may be revealed by assessing migration events, including geographical migration and evolutionary processes, each of which may be marked by genetic evidence [73]. In the present study, gene flow and divergence estimates further confirmed that extensive gene exchange may have occurred among Yarkand hare populations during ancient geological periods. According to geological evidence, previous Yarkand hare habitats were more continuous than current habitats [20]. Thus, migration events may have contributed to the southwest group’s AKT, WQ, and KS (three individuals) remaining in the north group lineage in large proportions. Similarly, the north group ALR population and some KRL individuals were clustered in the TX population lineage (*K* = 3). Notably, one of the three KS population migration events involved migration to the KRL population (Fig. 3b), which may be a reasonable explanation for the KS and KRL populations clustering together in our phylogenetic analysis (Fig. 2).

Two main explanations can be proposed for this relatively extensive gene flow between geographically isolated populations. The first possibility is related to the intrinsic features of the hare. Despite the harsh living conditions of the Tarim Basin, the Yarkand hare as a small mammal has strong adaptability to environmental changes. Furthermore, large effective population sizes, rapid locomotion, and extensive and long-distance dispersal capability can all promote gene exchange between populations along the oases, villages, farmlands, and fixed and semi-fixed sand dunes on the edge and surrounding the Gobi Desert. Moreover, gene exchange may be facilitated through the “green channels” that have been constructed for wildlife on roads and highways. Similarly, green corridors and bridges over rivers may also facilitate gene exchange. The second possibility is that gene flow has been maintained owing to repeated migration events toward glacial shelters under climate variations. A certain degree of gene exchange between the southwest populations in high-altitude areas near the Tarim Basin and the north populations at lower elevations in the basin’s hinterland may be related to refugia migration in the southwestern regions of the Tarim Basin during the Quaternary glacial period [15]. In general, areas with high biodiversity such as those maintaining stable habitats and accumulating genetic diversity during significant environmental changes are considered to provide biological refugia to species [71, 74]. Considering the overall high nucleotide diversity of the southwest group (Table 1), its root position in the phylogenetic tree (Fig. 2a), and the direction of desert expansion and shifting sand dunes, migration was likely concentrated from the northeast to the southwest. Moreover, the southwestern regions of the Tarim Basin, as the origin of rivers in the basin, may have acted as a glacial refugia for the Yarkand hare, a finding that is consistent with previous mitochondrial marker-based results [15]. Following the retreat of glaciers during the Penultimate Glacial Period (0.30–0.13 Mya) [75], species in glacial refugia likely recolonized the northern and eastern regions of the basin [15, 76]. Furthermore, with increasing glacial meltwater, rivers reoccupied their courses and oases were restored in the center of the basin due to the relatively warm and humid climate near the end of the Late Pleistocene (0.13– 0.07 Mya) [75, 77]. During recolonization, rivers may play a significant role in forming oases and green oasis corridors, along which hares could disperse, likely promoting extensive gene flow between the northern and southern populations [20]. Similar recolonization patterns were found in many European and North American species during ice ages [74]. As a third possibility, demographic and range expansions of Yarkand hare [8, 20, 78] might have supported extensive gene flow among populations.

The coexistence of genetic differentiation and gene flow of Yarkand hare populations is not a surprising result considering the environmental, geological, and evolutionary history. Climatic fluctuations in the Pleistocene as well as mountain and plateau uplift around the Tarim Basin are likely important factors in the differentiation and migration of basin hare populations. Based on high-quality SNP analysis, the most recent common ancestor of *Lepus yarkandensis* and *Lepus timidus* was estimated to have occurred approximately 0.86 Mya (Fig. 3a). This time interval is consistent with a period of desertification in the Tarim Basin, during which a drought climate and desert-like habitat began to dominate the entire basin [79, 80]. This occurred during the middle Pleistocene transition (approximately 1.25–0.70 Mya) [81] and following the formation of the Taklimakan Desert (approximately 5.3 Mya) [16, 82]. During this period, the hare ancestors gradually adapted to the dry environment of the basin, eventually evolving into the Yarkand hare. The divergence time of Yarkand hare (0.86 Mya) estimated herein is in agreement with the results obtained from mitochondrial genes (0.83 Mya [8]; 0.84 Mya [83]), combined with numerous accurate fossil datasets. Notably, this divergence time is similar to that of other species currently living around the Tarim Basin, including *Cervus elaphus yarkandensis* (0.8–2.2 Mya [73]; 0.98 Mya [84]), and with the timing of the most recent common ancestor *Phrynocephalus axillaris* (0.88 Mya) and the appearance of *Phrynocephalus forsythii* in the Tarim Basin (0.94 Mya) [85].

Divergence among the Yarkand hare populations may have resulted from glacial-induced fragmentation and follow-up recolonization during the early/middle Pleistocene. The KS population, located at the root of the phylogenetic tree with high genetic diversity, was estimated to have been the first Yarkand hare population to have diverged approximately 0.81–0.49 Mya (Fig. 3a), confirming its relatively older evolutionary history and further suggesting that the earliest Yarkand hare ancestor population may have inhabited the area near the Kashgar oasis in the southwest of the basin.

Approximately 0.880–0.5 Mya, rapid uplift of the Tibetan-Pamir Plateau as well as reduced air circulation strength and rainfall in the Tianshan Mountains further enhanced the aridity of the Tarim Basin and expanded the Taklimakan Desert [79]. Simultaneously, during the global ice maximum 0.7–0.6 Mya, maximum glaciation occurred in the Tibetan Plateau, likely reducing the amount of water vapor conveyed by the westerly winds to the Tarim Basin, resulting in extreme desert expansion [86, 87]. Hare populations in the basin would have had to spread toward the refugia to develop suitable habitats, which match the differentiation time of the southwestern populations 0.8–0.49 Mya (Fig. 3a). Divergence events of other populations occurred approximately 0.46–0.32 Mya (Fig. 3a), during the great interglaciation period (0.5–0.3 Mya) of the Pleistocene, where the basin climate was likely warmer and moister than that in the former sub-stage [75, 88]. As temperatures increased, the river systems along the edge of the Tarim Basin were flooded with greater amounts of runoff from melting snow and glacial ice [89]. Importantly, mesophytic herbaceous plants in the basin were widely distributed 0.4–0.25 Mya [88], which would be effective at dispersing hares toward the outside of the refugia, thereby contributing to the evolution and formation of northern populations (0.36–0.34 Mya) (Fig. 3a).

Subsequently, during the Penultimate (0.3–0.13 Mya) and Yurunkax glaciation (0.333 ± 0.046 Mya) [90] of the West Kunlun Mountains [75, 90], the basin climate became increasingly cold and arid, with a significantly reduced amount of melting glacial snow in the summer that contributed to the drying up of rivers. This resulted in the shrinkage or fragmentation of the original habitat of Yarkand hares, causing their retreat toward the southwestern refugia.

Based on our results and previous studies [15, 19, 20], the pattern of historical gene flow and divergence times of the southwest and north group Yarkand hare populations can be hypothesized as follows: the species originated from the southwestern parts of the Tarim Basin, expanding north during interglacial periods and contracting to the southwestern areas during glacial periods. Such repeated differentiation and recolonization events may have promoted the range expansion of the Yarkand hares, followed by extensive gene flow among populations. However, due to limited Tarim Basin sampling sites, our prediction regarding the migration of Yarkand hare involves only the southwestern and northern regions of the Tarim Basin, which have been recognized as the key areas for glacial refuge and migration events based on previous mtDNA results [15]. Therefore, the generalization of our predictions for the entire range of the species must be verified by including southeast samples in similar analyses in the future.

### Unique characteristics of the TX population

In contrast to other samples from the oases or mountains around the basin, samples from the TX population were collected from the Pamir Plateau in the upper reaches of Yarkand River, near the southwestern region of the Tarim Basin. Surprisingly, all TX samples were completely separated from the southwest group in PCA and instead clustered with north group samples in the phylogenetic tree (Fig. 2). ADMIXTURE Bayesian clustering analysis also showed that the TX population was independent of a pure ancestor when *K* = 3. In addition, all individuals from the ALR population in the north group, as well as some KRL individuals, had large proportions of samples in the TX lineage (*K* = 3, Fig. 2c). Our results suggest that patterns of admixture in TX, ALR, and KRL populations between the southwestern and north groups may have resulted from migration events (Fig. 3b). Furthermore, the low pairwise *F*_ST_ values between the TX and north group populations (Table 2) indicated their close phylogenetic relationship, suggesting that they may have originated from the same ancestral migration population and insufficient time has passed for their divergence, despite their current geographical isolation [30]. These migration events were further verified using TreeMix analyses (Fig. 3b). Considering the above results and those of previous studies [8, 15, 19], we deduced that during Penultimate Glaciation (0.3–0.13 Mya), the habitats of the north group population were fragmented due to river disruption and oasis shrinkage caused by cold and dry weather along with reduced glacial meltwater. One of the ancestral migration populations was estimated to have diverged from the north populations 0.32 Mya (Fig. 3a), initially spreading from the southwestern regions of the Tarim Basin during the interglacial period and then returning to the southwestern refuge during the second stage of the Penultimate Glacial Period (0.277–0.266 Mya) in the West Kunlun Mountains [91]. During the southwestern dispersal, some hares migrated upstream and dispersed alongside Yarkand River, eventually arriving at Tashkurgan County, where they formed the TX population. Ultimately, the climate became colder and drier during the ice age, and this population adapted to the distinct plateau environment, remaining isolated from other southwest hare populations.

### Selection signatures in Yarkand hare populations

The Yarkand hare is a typical drought-tolerant hare endemic to the Tarim Basin around the Taklimakan Desert in Xinjiang, China. In particular, this species has adapted to extreme aridity, intense solar radiation, large diurnal temperature variations, and hot environments [15]. To characterize the regions with environmental differences among the sampled Yarkand hare populations, we conducted selective sweep analysis and identified several candidate genes with a high degree of differentiation (Additional file 5: Table S2).

Different genes selected in the north [e.g., F-box and WD repeat domain containing 8 (*FBXW8*)] and southwest [e.g., aryl hydrocarbon receptor nuclear translocator like (*ARNTL*), heat shock protein 90 beta family member 1 (*HSP90B1*), and ubiquitination factor E4B (*UBE4B*)] groups were significantly enriched in the same catabolic and metabolic processes, as well as in the corticosteroid and glucocorticoid receptor signaling pathways; these genes were also commonly enriched in development-related GO terms (Additional file 6: Table S3). These candidate genes and their associated biological processes indicate the importance of energy supplementation for the Yarkand hare to adapt to extreme drought environments with saline–alkaline soil and poor food resources. A similar assumption was made based on whole-genome sequencing data of Tarim red deer (*C.e. yarkandensis*) and sheep breeds (*Ovis aries*) indicating adaptation to extreme drought environments [84, 92]. Two candidate genes, laminin subunit beta 1 (*LAMB1*) and integrin subunit alpha 1 (*ITGA1*), selected in the southwest group were significantly enriched in pathways related to cell survival and proliferation, including ECM-receptor interaction (KEGG pathway accession code: ocu04512), PI3K-AKT signaling (KEGG pathway accession code: ocu04151), and focal adhesion pathway (KEGG pathway accession code: ocu04510) (Additional file 6: Table S3). These pathways are explicitly associated with responses of the lung, heart, and spleen of yak to altered elevation, and have been shown to play a pivotal role in the adaptation of yak to hypoxia [93]. In addition to arid adaptation, the southwest populations of the Yarkand hare also live at higher altitudes (> 1500 m above sea level) than those in the north. Therefore, we speculated that these pathways and related candidate genes may explain the potential molecular mechanisms underlying the adaptation of southwest Yarkand hare populations to hypobaric hypoxia in medium-altitude areas. This suggests that specific factors influencing natural selection may act on similar functional biological pathways in different species, driving their adaptation to the same environments.

We identified 17 candidate genes via putative selection sweeps between north populations and the TX population, only three of which were selected in the north group. The biological processes and pathway functions of the other 14 genes selected in the TX population indicate that the special environment may have forcibly shaped the genomic differentiation in this population (Additional file 7: Table S4); this may be associated with survival of the Yarkand hare in a cold, arid, and high-altitude environment. For instance, the candidate gene polycystin- 2, transient receptor potential cation channel (*PKD2*) selected in TX encodes an integral membrane glycoprotein [94] that is similar to calcium channel subunits and is required for the development of a normal renal tubular architecture [95]. *PKD2* was significantly enriched in several GO biological process terms, including kidney and renal system-related morphogenesis and development, sodium channel activity, response to water stimulus, and response to osmotic stress (Additional file 7: Table S4). All of these GO terms are functionally related to regulating water reabsorption, renal cell metabolism, and blood vessels in the kidney, and may therefore enable the Yarkand hare TX population to reabsorb water more efficiently in an arid environment. *PKD2* and three other genes selected in TX [ALK receptor tyrosine kinase (*ALK*), fibrillin 2 (*FBN2*), and α-kinase anchoring protein 6 (*AKAP6*)] were significantly associated with responses to various stimuli (eight GO terms, *p* < 0.05; Additional file 7: Table S4), indicating that these genes and GO terms may be functionally related to hypoxia responses in the plateau environment of TX. Notably, another candidate selected gene in TX, cytochrome P450, family 4, subfamily A, polypeptide 5 (*CYP4A5*), was significantly enriched in the KEGG pathways fatty acid degradation (KEGG pathway accession code: ocu00830), retinol metabolism (KEGG pathway accession code: ocu05223), and arachidonic acid metabolism pathway (KEGG pathway accession code: ocu00590). *CYP4A5* plays an important role in converting arachidonic acid into 19(S)-HETE and 20-HETE through ω–hydroxylation (Additional file 7: Table S4). 19(S)-HETE has been reported as a potent vasodilator of renal preglomerular vessels that stimulate water reabsorption [96]. Interestingly, the arachidonic acid metabolism pathway is a key molecular mechanism underlying mammalian adaptation to arid deserts [84, 92, 97]. 20-HETE substantially affects renal tubular and vascular function [98] by regulating vasodilation and contraction, as well as by promoting endothelial cell proliferation and angiogenesis. The balance between vascular contraction and relaxation plays a pivotal role in hypoxia-induced pulmonary arterial hypertension [99] and congestive heart failure [100]. Therefore, we speculate that the candidate gene *CYP4A5* may play an essential role in survival of the Yarkand hare TX population in arid and altitude environments. Nevertheless, the specific roles of these candidate genes require further validation.

Besides drought stress, intense solar radiation is another major environmental factor affecting the survival of Yarkand hares in the Tarim Basin under altitude-related hypoxic conditions. Chronic exposure to solar UV radiation is a potent inducer of reactive oxygen species (ROS). Oxidative damage is induced by oxidative stress when ROS formation exceeds the antioxidant defense capacity of target cells and is associated with various disease states [101]. The candidate genes identified in the southwest group, such as oxidation resistance 1 (*OXR1*), and in the TX population, such as leucine rich repeat kinase 2 (*LRRK2*), were significantly enriched in 18 GO terms related to the regulation and response to oxidative stress and peroxidase activity (Additional file 8: Table S5); this indicates that under stress conditions, these genes protect cells from oxidative stress-induced damage [102, 103]. Such regulation of the cellular stress response may play an important role in the adaptation of these populations to harsh environments. Furthermore, the candidate gene dedicator of cytokinesis 7 (*DOCK7*) selected in TX is involved in the regulation of smooth muscle cell and vascular-associated smooth muscle cell migration (Additional file 8: Table S5). *LRRK2* and *DOCK7* were also significantly enriched in five biological process terms (GO: 0008021, GO: 0031398, GO: 0010038, GO: 0017016, and GO: 0006979, *p* < 0.05; Additional file 8: Table S5) that were previously identified as lineage-specific processes associated with the adaptation of yak to high altitudes [104]. Accordingly, we assumed that *LRRK2* and *DOCK7* may have potential roles in the adaptation of Yarkand hare populations to cold and high-altitude environments. Nevertheless, these functions need to be confirmed by functional analysis of the identified genes with traits of adaptive evolution in comparative stress studies of Yarkand hare and other animals living at high altitudes.

Overall, we detected only a small number of candidate genes (58) within selected regions among Yarkand hare populations, which may be attributed to the limited sampling sites or the small and unequal sample sizes for the southwest group and TX population. Moreover, the reduced-representation sequencing approach—which may not detect low-frequency alleles that could have a substantial role in adaptation— exhibits some inherent limitations with respect to obtaining information regarding neutral or adaptive genetic variation on a genome-wide scale [29]. Because of these limitations, we have not considered more stringent settings for *p*-values, such as Bonferroni correction, false discovery rate, and fold enrichment tests. Nonetheless, we identified noteworthy, overrepresented biological processes and pathways based on raw *p*-values, which may provide insights into the mechanisms involved in the environmental adaptation of this species. It is also important to note that many evolutionary processes can influence genomic variation. Without functional experiments, it is difficult to distinguish natural selection from genetic drift at a particular locus, which includes the candidate genes identified herein. Therefore, comprehensive studies integrating whole-genome re-sequencing, demographic history, candidate gene sequencing, and transcriptomes with adaptive traits and physiological mechanisms of Yarkand hare are warranted to confirm or refine our functional genomic hypotheses and further explore the genomic mechanisms underlying adaptation.

## Conclusions

We performed SNP analysis via SLAF-seq to elucidate the genetic diversity, differentiation, phylogenetic relationships, historical pattern of divergence, and gene flow among seven Yarkand hare populations in the southwestern and northern regions of the Tarim Basin at the genome-wide level. We also conducted selective sweep analysis to assess differences in the SNP distribution across chromosomes and determine their associations with environmental stress or adaptation.

Our genomic data revealed a relatively high degree of genetic diversity along with a high degree of admixture between populations through multiple analyses, although there was a clear population structure and low to moderate differentiation between the southwest and north groups. The coexistence of genetic differentiation and admixture among the Yarkand hare populations sampled in this study likely reflects the strong adaptability and enhanced dispersal characteristics of this species, as well as the important influence of historical and current environmental changes. We further investigated the selection signatures that may be caused by different types of environmental selective pressure, and identified genes and pathways that may be related to drought environment adaptation of the Yarkand hare. We also screened genes and pathways likely associated with altitude adaptation of the TX and southwest populations. Overall, this study highlights the value of genomic data in assessing the population genetics of this endemic species, thereby prompting future gene linkage and association analyses of key adaptive traits linked to the specific habitats of these and other hare populations.

## Supplementary Information

**Additional file 1: Table S1**. Summary of Yarkand hare sequencing data obtained in this study.

**Additional file 2: Figure S1**. Distribution of SLAF tags on reference genome chromosomes. The abscissa represents the SLAF tag position on the chromosome and the ordinate represents each chromosome. The reference genome was divided into 1-Mb portions. The higher the number of SLAF tags per 1 Mb, the darker the color; the lower the number, the lighter the color.

**Additional file 3: Figure S2**. Phylogenetic trees constructed using ML (a) and BI (b) methods based on the SNP matrix of 76 Yarkand hares. *Oryctolagus cuniculus* was used as the outgroup. AKS, Akesu; ALR, Alar; KRL, Korla; TX Taxkorgan; AKT, Aketu; KS, Kashgar; WQ, Wuqia.

**Additional file 4: Figure S3**. Cross-validation errors in the ADMIXTURE analysis. The number of ancestry (*K*) was assumed to range from 1 to 10; K = 2 was the optimal number.

**Additional file 5: Table S2**. List of genes in the overlapping regions selected by the top 5% highest log_2_ (*θ*_π_ ratio) and top 5% highest *F*_ST_ values for Yarkand hare populations from different environments.

**Additional file 6: Table S3**. The top 20 KEGG pathways and GO functional enrichment of candidate genes between the north and southwest groups via *F*_ST_-*θ*_π_ ratio analysis.

**Additional file 7: Table S4**. KEGG pathways and GO functional enrichment of candidate genes between the north group and TX population via *F*_ST_-*θ*_π_ ratio analysis.

**Additional file 8: Table S5**. KEGG pathways and GO functional enrichment of candidate genes between the southwest group and TX population via *F*_ST_-*θ*_π_ ratio analysis.

## Data Availability

The data that support the findings of this study have been deposited into CNGB Sequence Archive (CNSA) [105] of China National GeneBank DataBase (CNGBdb) [106] with accession number CNP0001925. The sequence data in our study has been uploaded to NCBI/Genbank with the accession number of PRJNA750896 and can be accessed under: https://www.ncbi.nlm.nih.gov/sra/PRJNA750896.
