# Supplementary material for: Revealing the coexistence of differentiation and communication in an endemic hare, Lepus yarkandensis (Mammalia, Leporidae) using specific-length amplified fragment sequencing
Source: Front Zool. 2021 Sep 26;18:50. doi: 10.1186/s12983-021-00432-x (PMC8474959; doi:10.1186/s12983-021-00432-x)
Supplement: Supplementary file 2 — Additional file 2: Figure S1. Distribution of SLAF tags on reference genome chromosomes. The abscissa represents the SLAF tag position on the chromosome and the ordinate represents each chromosome. The reference genome was divided into 1-Mb portions. The higher the number of SLAF tags per 1 Mb, the darker the color; the lower the number, the lighter the color. [file 12983_2021_432_MOESM2_ESM.pdf]

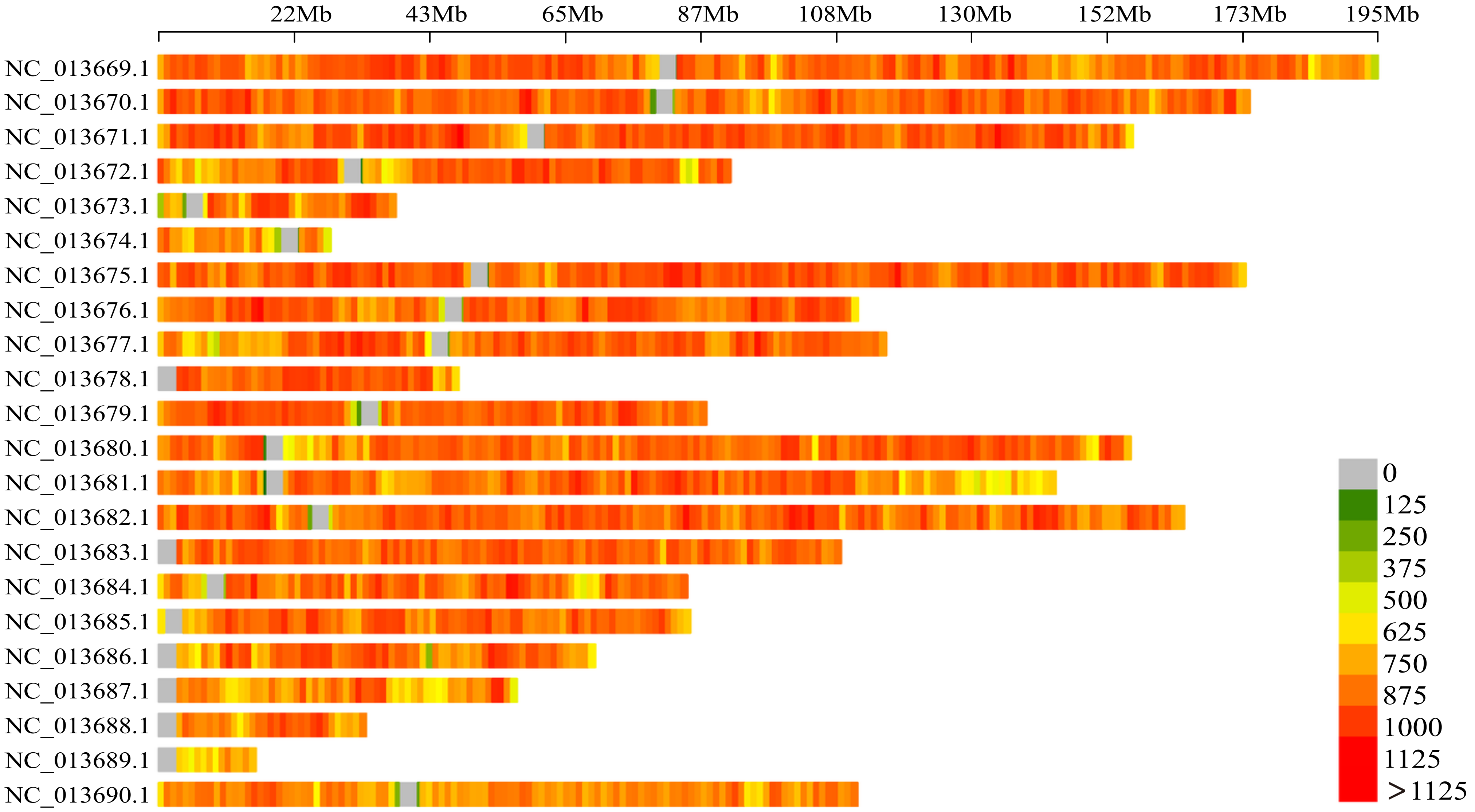

Additional file 2: Fig S1. Distribution of SLAF tags on reference genome chromosomes. The abscissa represents the SLAF tag position on the chromosome and the ordinate represents each chromosome. The reference genome was divided into 1-Mb portions. The higher the number of SLAF tags per 1 Mb, the darker the color; the lower the number, the lighter the color.
