# Supplementary material for: Revealing the coexistence of differentiation and communication in an endemic hare, Lepus yarkandensis (Mammalia, Leporidae) using specific-length amplified fragment sequencing
Source: Front Zool. 2021 Sep 26;18:50. doi: 10.1186/s12983-021-00432-x (PMC8474959; doi:10.1186/s12983-021-00432-x)
Supplement: Supplementary file 3 — Additional file 3: Figure S2. Phylogenetic trees constructed using ML (a) and BI (b) methods based on the SNP matrix of 76 Yarkand hares. Oryctolagus cuniculus was used as the outgroup. AKS, Akesu; ALR, Alar; KRL, Korla; TX Taxkorgan; AKT, Aketu; KS, Kashgar; WQ, Wuqia. [file 12983_2021_432_MOESM3_ESM.pdf]

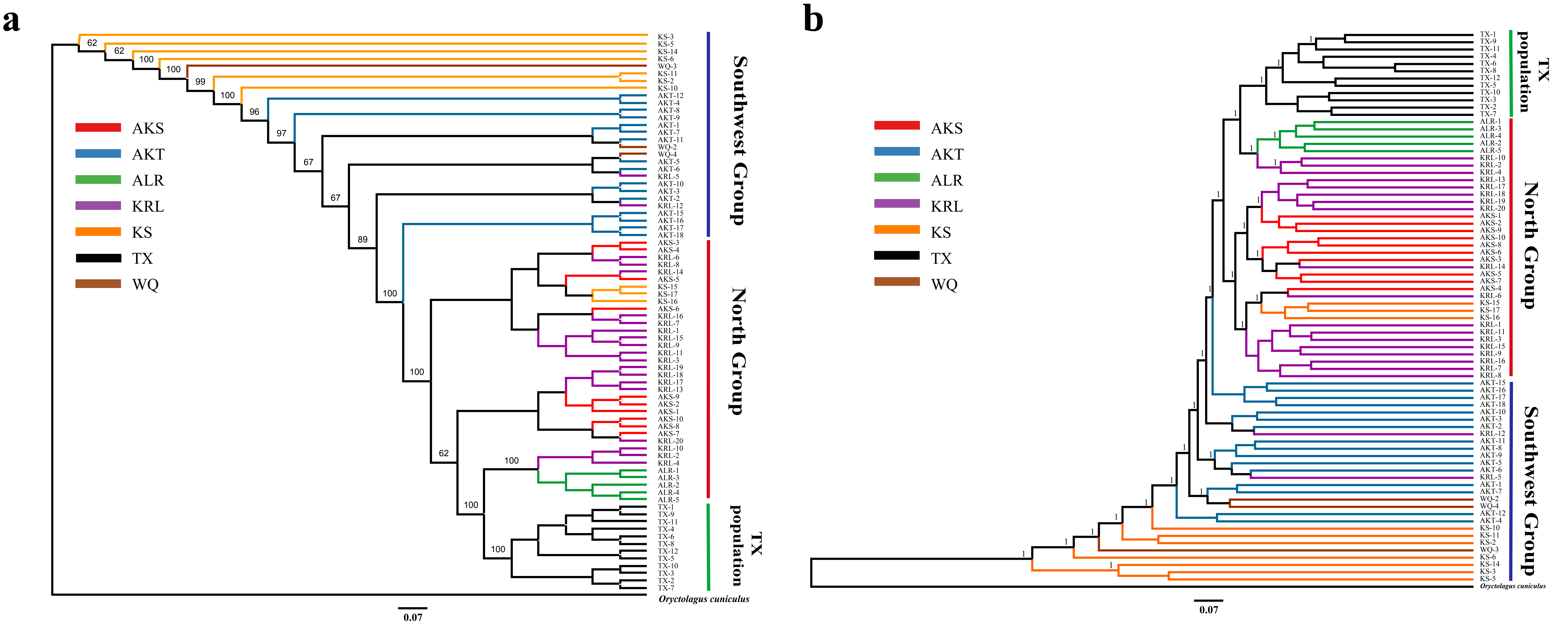


**Additional file 3: Fig S2**. Phylogenetic trees constructed using ML (a) and BI (b) methods based on the SNP matrix of 76 Yarkand hares. *Oryctolagus cuniculus* was used as the outgroup. AKS, Akesu; ALR, Alar; KRL, Korla; TX Taxkorgan; AKT, Aketu; KS, Kashgar; WQ, Wuqia.
