# Supplementary material for: Revealing the coexistence of differentiation and communication in an endemic hare, Lepus yarkandensis (Mammalia, Leporidae) using specific-length amplified fragment sequencing
Source: Front Zool. 2021 Sep 26;18:50. doi: 10.1186/s12983-021-00432-x (PMC8474959; doi:10.1186/s12983-021-00432-x)
Supplement: Supplementary file 4 — Additional file 4: Figure S3. Cross-validation errors in the ADMIXTURE analysis. The number of ancestry (K) was assumed to range from 1 to 10; K = 2 was the optimal number. [file 12983_2021_432_MOESM4_ESM.pdf]

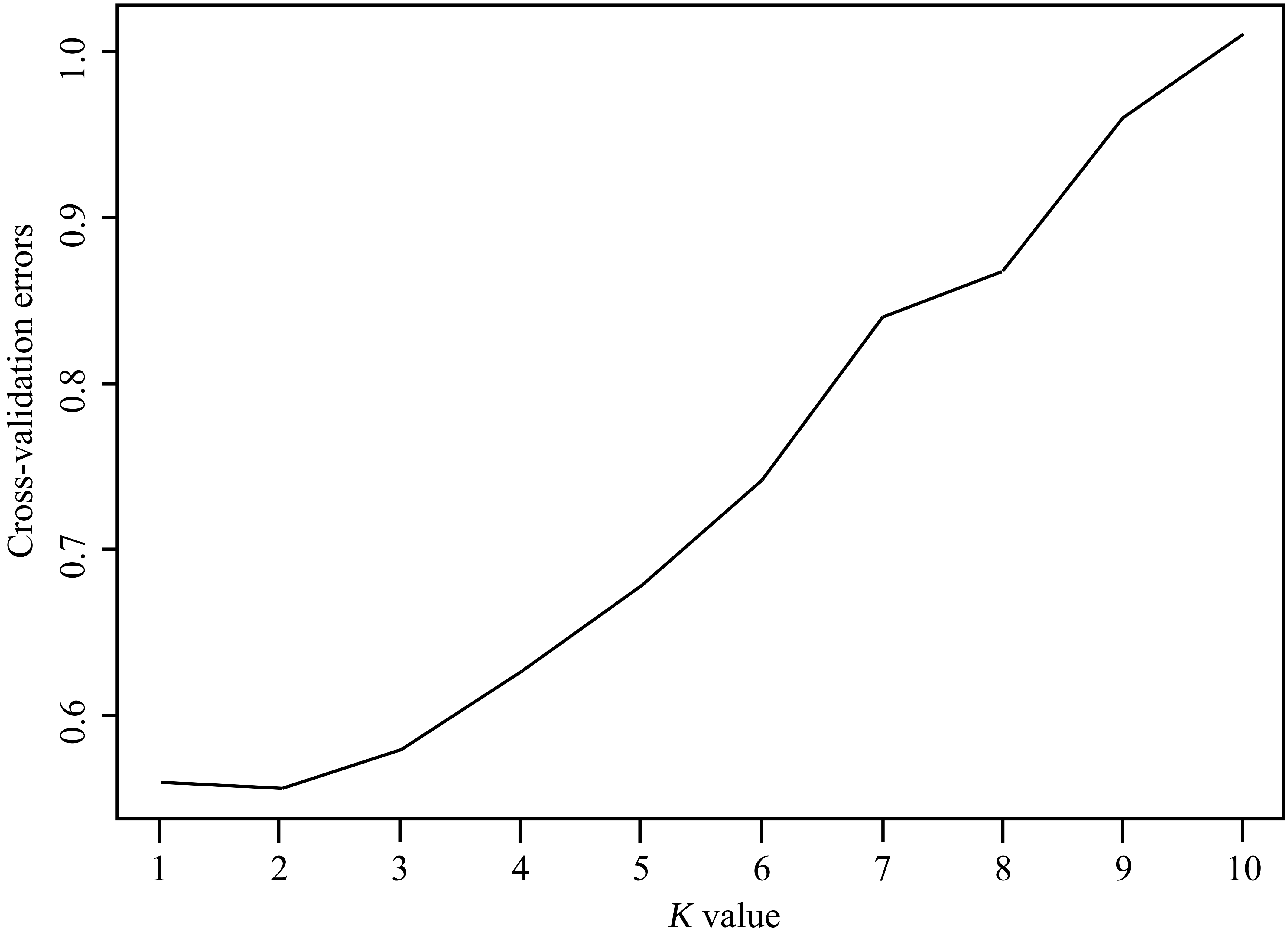

Additional file 4: Fig S3. Cross-validation errors in the ADMIXTURE analysis. The number of ancestry (*K*) was assumed to range from 1 to 10; *K* = 2 was the optimal number.
